# A deep learning and radiomics fusion model enhances endoscopic ultrasonography diagnosis of gastric tumors

**DOI:** 10.3389/fonc.2026.1718061

**Published:** 2026-05-07

**Authors:** Yi Liu, Jinpeng Li, Yuan Ning, Yanjun Wu, Peiyuan He, Xianling Dong

**Affiliations:** 1 Department of Biomedical Engineering, Chengde Medical University, Chengde, China; 2 Department of Gastroenterology, Affiliated Hospital of Chengde Medical University, Chengde, China

**Keywords:** clinical features, deep learning, endosonography, gastric tumors, radiomics

## Abstract

**Objectives:**

Gastric tumors are one of the most common digestive system diseases, and their accurate diagnosis largely depends on the expertise of medical professionals in interpreting imaging data. As medical imaging technology has advanced, the volume of imaging data for gastric tumors has rapidly increased. This wealth of data provides valuable diagnostic information but also poses challenges for experts in diagnosis and analysis. To address these challenges, a more accurate and efficient diagnostic model is urgently needed.

**Methods:**

In this study, we propose a gastric tumor classification model that combines deep learning and radiomic features to differentiate gastrointestinal stromal tumors from other gastric tumor types. This retrospective study collected 4,806 endoscopic ultrasound images from 219 patients. Deep learning and radiomic features were extracted, and feature selection was performed using t-tests and the LASSO method. Additionally, clinical features were recorded. Single-parameter models were constructed based on these three categories of features. Following this, a gastrointestinal tumor classification model was developed by integrating the predictions from the single-parameter models.

**Results:**

The model achieved an area under the receiver operating characteristic curve of 0.95, with sensitivities, specificities, positive predictive values, negative predictive values, and accuracies of 100.00%, 82.86%, 60.00%, 100.00%, and 86.36%, respectively.

**Conclusions:**

The experimental results indicate that the gastric tumor classification model has demonstrated strong performance in classifying gastric tumors using endoscopic ultrasound images, offering valuable diagnostic support for clinicians.

## 1 Introduction

Nearly two million people die annually from gastrointestinal disorders (1). Gastric tumors, as one of the most prevalent conditions, garner significant attention in the medical field regarding their diagnosis and treatment. Gastrointestinal stromal tumors (GISTs) are the most common mesenchymal tumors of the gastrointestinal tract, originating from the submucosa. Obtaining conclusive pathology through routine preoperative gastroscopic biopsy can often be challenging. GISTs need to be differentiated from other gastric submucosal tumors, such as smooth muscle tumors, nerve sheath tumors, lipomas, and ectopic pancreas. Although the imaging manifestations of these tumors can be similar to GISTs, their biological behaviors, prognoses, and treatments differ significantly (2). This highlights the critical importance and urgency of performing a preoperative differential diagnosis for GISTs. Accurately differentiating GISTs from other benign or low-risk lesions is essential for determining the appropriate clinical approach, such as whether to proceed with surgical resection or opt for conservative follow-up. Addressing this issue reflects a significant unmet clinical need.

In clinical practice, accurately diagnosing GISTs requires comprehensive imaging studies, immunohistochemical analysis, and pathological examinations. Early diagnosis is crucial for achieving high rates of disease-free survival. However, these extensive tests are time-consuming. Therefore, implementing technology that can predict GISTs may significantly improve the overall prognosis. Endoscopic ultrasonography plays a vital role in the early diagnosis of gastrointestinal tumors. The accurate diagnosis of gastric tumors heavily relies on the experience and judgment of healthcare professionals when interpreting imaging features. This reliance contributes to inter-observer variability and inconsistencies in diagnoses, which is a significant limitation of current endoscopic ultrasound (EUS) as a standalone diagnostic tool. As a result, there is a strong motivation for researchers to develop more objective and reliable predictive methods.

Currently, deep learning technology plays a crucial role in achieving this objective, demonstrating great potential and diverse applications in gastrointestinal imaging. For example, researchers have created frameworks such as the Spectrum-Aided Vision Enhancer (SAVE), which successfully transforms white-light endoscopic images into simulated hyperspectral and narrow-band imaging data. This methodology has greatly improved the performance of deep learning models in classifying a range of gastrointestinal diseases, including polyps, esophagitis, and ulcerative colitis (3–5). These advancements validate the effectiveness of deep learning models in processing and interpreting complex endoscopic image information.

With advancements in technology, convolutional neural networks (CNNs) have become widely utilized in various fields, demonstrating promising results, particularly in disease classification. This includes the identification of gastric tumors (6, 7), pancreatic masses (8), and plasma cystic tumors (9). However, in clinical practice, CNNs face limitations due to their requirement for large-scale datasets to effectively train the models. Collecting such extensive data for specific clinical tasks can be quite challenging. As a result, some studies have opted to use pre-trained CNNs as feature extractors to derive medical image features. Unfortunately, this approach often overlooks critical global features, such as size and volume, which are important in clinical contexts. This oversight can lead to decreased classification performance. Therefore, it becomes evident that single-modality deep learning methods, which rely solely on end-to-end learning, are constrained in clinical scenarios where data is limited and explicit quantitative features are essential.

In clinical practice, radiomics is widely used because it can be applied to various types of medical images. Numerous studies have shown that radiomics is effective in classifying tumors, including breast tumors (10, 11), colorectal tumors (12), gastric tumors (13), hepatocellular carcinoma (14, 15), and retroperitoneal sarcoma (16), often yielding impressive results. However, radiomic features are typically derived from relatively global regions of interest, and the specificity of the features for particular diseases is not always thoroughly examined. As a result, valuable local features that could enhance classification performance may go unnoticed. Radiomics methods that rely solely on handcrafted features are insufficient for capturing the most relevant and distinctive deep image patterns related to the disease.

In summary, while both CNNs and radiomics models demonstrate strong performance in disease classification, they each have inherent limitations as “single-modality” solutions for the specific clinical need of differentiating GIST using EUS images. CNNs may struggle to interpret and utilize clinically important quantitative features, whereas radiomics may not effectively learn the optimal image representations. The complementary capabilities of these two models inspire researchers to integrate their strengths for more satisfactory results. The introduction of deep learning features enables radiomics to obtain intricate structures related to specific tasks, leading to excellent performance in tumor characterization and prognostic prediction for various conditions, including intracranial aneurysm (17), gastric (18–26), breast cancer (27), lung nodules (28), cystic renal lesions (29), and nasopharyngeal cancers (30). Studies have demonstrated that combining deep learning models with radiomics is a viable approach for medical image classification.

Currently, there is not enough evidence to support the specialized integration of deep learning and radiomics techniques for classifying gastric tumors based on endoscopic ultrasound images. This integration aims to address the critical need for preoperative diagnostics. In this study, we intend to optimize the strengths and address the limitations of both radiomics and CNNs by developing and testing a hybrid model. This model is designed to automatically differentiate between GISTs and non-GISTs.

The main contributions of this paper are as follows. First, we propose a multi-modal feature fusion framework that integrates deep learning features, radiomic features, and clinical parameters to characterize gastric tumors in EUS images, achieving satisfactory results. Second, we introduce a deep learning-radiomics integration framework designed to enhance the diagnostic accuracy of EUS for gastric tumors by specifically differentiating GISTs from other gastric tumor types. This addresses a pressing clinical challenge associated with the growing volume of medical imaging data. Third, the proposed model achieves outstanding performance, with an Area Under the ROC Curve (AUC) of 0.95 and an accuracy of 86.36%, demonstrating its potential as a powerful tool for accurate diagnosis. Evaluation shows that our method improves the precision of EUS image classification and surpasses recent state-of-the-art results. Finally, the model serves as an effective tool to assist medical professionals in interpreting complex imaging data, thereby potentially increasing diagnostic efficiency and offering valuable support for clinical decision-making.

The remainder of the paper is organized as follows. Section 2 reviews related works of endoscopic ultrasound image classification. Section 3 presents the proposed method. Section 4 presents the experimental results and their analysis, followed by the conclusions in Section 5.

## 2 Related works

Gastrointestinal stromal tumors demonstrate an annual incidence of approximately 1.2 per 100,000 population across most geographical regions (2). Histopathological diagnosis, while initially guided by tumor morphology, requires definitive confirmation through immunohistochemical analysis. The KIT (CD117) protein serves as the principal diagnostic marker, exhibiting positivity in 95% of GIST cases. Notably, KIT immunoreactivity alone lacks specificity as it may also be present in metastatic melanoma, angiosarcoma, and certain pediatric malignancies, necessitating comprehensive evaluation of both architectural and immunophenotypic characteristics. In clinical practice, pathological biopsy remains the diagnostic gold standard, supplemented by preoperative imaging modalities including contrast-enhanced CT and endoscopic ultrasound. Although EUS provides superior spatial resolution for submucosal lesion characterization, its diagnostic accuracy demonstrates significant operator dependence and interobserver variability. Furthermore, sampling limitations during biopsy procedures may yield false-negative results, potentially compromising timely therapeutic intervention.

Recent advances in deep learning have demonstrated significant progress in the diagnosis and prediction of the malignant potential of gastrointestinal stromal tumors. Convolutional neural network models based on endoscopic ultrasound and CT images have been successfully applied for differential diagnosis between GISTs and other submucosal tumors, as well as malignant potential assessment. For tumor classification, the CNN model developed by Seven et al. (6) achieved 86.98% accuracy in distinguishing GISTs from leiomyomas on 384 validation images, outperforming endoscopy experts’ 63% accuracy. Oh et al. (18) employed an EfficientNet architecture, which showed exceptional performance in patient-level analysis, with 100% sensitivity, 85.7% specificity, and 96.3% negative predictive value. The systematic review by Liu et al. (19), which included data from eight studies, reported a pooled sensitivity of 93% (95% CI 0.85-0.96) and a specificity of 88% for differentiating between AI-based GIST and leiomyoma. In malignant potential prediction, Zhuo et al. (20) developed an ultrasound-based ResNet18 model achieving 88% accuracy, significantly surpassing radiologists’ performance. Wang et al. (21) proposed a venous-phase CT CNN model that demonstrated consistent diagnostic performance with an AUC of 0.86 for high-risk GIST prediction and maintained good discrimination across different risk levels (AUC 0.89-0.90 for low-risk). Notably, Grad-CAM visualization revealed that models primarily focus on key imaging features, such as cystic necrosis and tumor margins (20). However, current deep learning models face several limitations including substantial data requirements (980 ultrasound images in (20), 376 EUS images in (21)), and insufficient integration of clinical parameters such as tumor location and patient age as noted in (19), which may constrain their clinical translation and warrant future improvements in data efficiency, model interpretability, and multimodal information fusion.

Radiomics has demonstrated significant potential in GIST risk stratification and molecular prediction by quantifying tumor heterogeneity through advanced image analysis. For risk assessment, Zhuo et al. (26) developed an ultrasound radiomics nomogram, achieving an AUC of 0.90 for predicting malignant potential, which outperformed clinical models by selecting 17 robust features from 1,306 candidates. Song et al. (31) further enhanced prediction accuracy by combining multiphase CT radiomics with clinical indicators, elevating AUC from 0.895 to 0.927 in multicenter validation. Molecular profiling studies revealed radiomics’ capability in genotype prediction, as demonstrated by Zhang et al. (32), whose CT-based model predicted KIT exon 11 deletions with AUC 0.863, showing significant prognostic value for recurrence (C-index = 0.864, p = 0.0088). Xie et al. (33) confirmed this approach in a multi-center study of 872 patients, where radiomics features improved D842V mutation detection (F1 score 0.253 vs 0.155, p = 0.012). However, limitations persist, including feature redundancy (only 7.8% of features showed malignancy correlation, p<0.01) and inter-observer variability in manual segmentation (Wang et al. (34) reported ICC<0.75 for 22% features). Xiao et al. (35) integrated radiomics with clinical parameters, and the model, which combined CT radiomics and pathomics, achieved superior RFS prediction C-index = 0.917 compared to unimodal approaches (p = 0.0088).

Multimodal fusion technology has demonstrated significant progress in tumor imaging diagnosis, with numerous studies confirming its superior diagnostic performance across various cancer types. In laryngeal carcinoma research, Wang et al. (36) developed the DLRad_DB model integrating 3D/2D deep learning and radiomics features, achieving an AUC of 0.89-0.90 for predicting occult lymph node metastasis. Similarly, Zhang et al. (37) established a multiparametric MRI-based deep learning radiomics model that showed excellent performance in glioma differentiation with an AUC of 0.947. Both CNNs and radiomics models have demonstrated strong capabilities in disease classification; however, each approach has inherent limitations. This complementary relationship has motivated researchers to combine their respective strengths for improved outcomes. The incorporation of deep learning enables radiomics to capture task-specific complex structures, leading to enhanced performance in tumor characterization and prognosis prediction across various malignancies, including intracranial aneurysms (17), gastric cancer (22–25), breast cancer (27), pulmonary nodules (28), cystic renal lesions (29), and nasopharyngeal carcinoma (30). These findings collectively support the feasibility of integrating deep learning with radiomics for medical image classification. However, limited evidence exists regarding the specific application of combined deep learning and radiomics techniques for gastric tumor classification using EUS images. Current research in GISTs has primarily focused on the fusion of CT and MRI modalities. For instance, Xiao et al. (35) developed a CT-pathomics multimodal model achieving a C-index of 0.917 for prognosis prediction, while Yang et al. (38) reported an MRI-based hybrid model with an AUC of 0.930 for mitotic index prediction.

EUS is an important diagnostic tool for gastric tumors, yet it remains underexplored in multimodal fusion studies. Although Gu et al. (39) demonstrated the potential of combining EUS with deep learning for pancreatic tumor diagnosis, there has been a lack of systematic research applying similar methods to gastric tumors. Furthermore, recent studies by Gandhi et al. (40–43) have successfully implemented deep learning and radiomics fusion with modalities such as MRI for Alzheimer’s disease and fundus photography or OCT for glaucoma. However, these applications differ significantly from EUS. Unlike MRI or OCT, which generally provide high-resolution, standardized, and low-noise images, EUS images are characterized by inherent speckle noise, a low contrast-to-noise ratio (CNR), a limited field of view, and a high degree of operator dependency. As a result, fusion strategies designed for high-quality imaging modalities cannot be directly applied to EUS without specific adaptations to address these challenges. To date, no study has systematically tackled these EUS-specific issues within a deep learning and radiomics fusion framework for gastric tumor classification. In this study, we aim to bridge this gap by integrating deep learning features, radiomics features, and clinical characteristics specifically tailored to EUS images. This approach will help establish a robust classification model for gastric tumors.

## 3 Methods

### 3.1 Method framework

**Figure 1:**
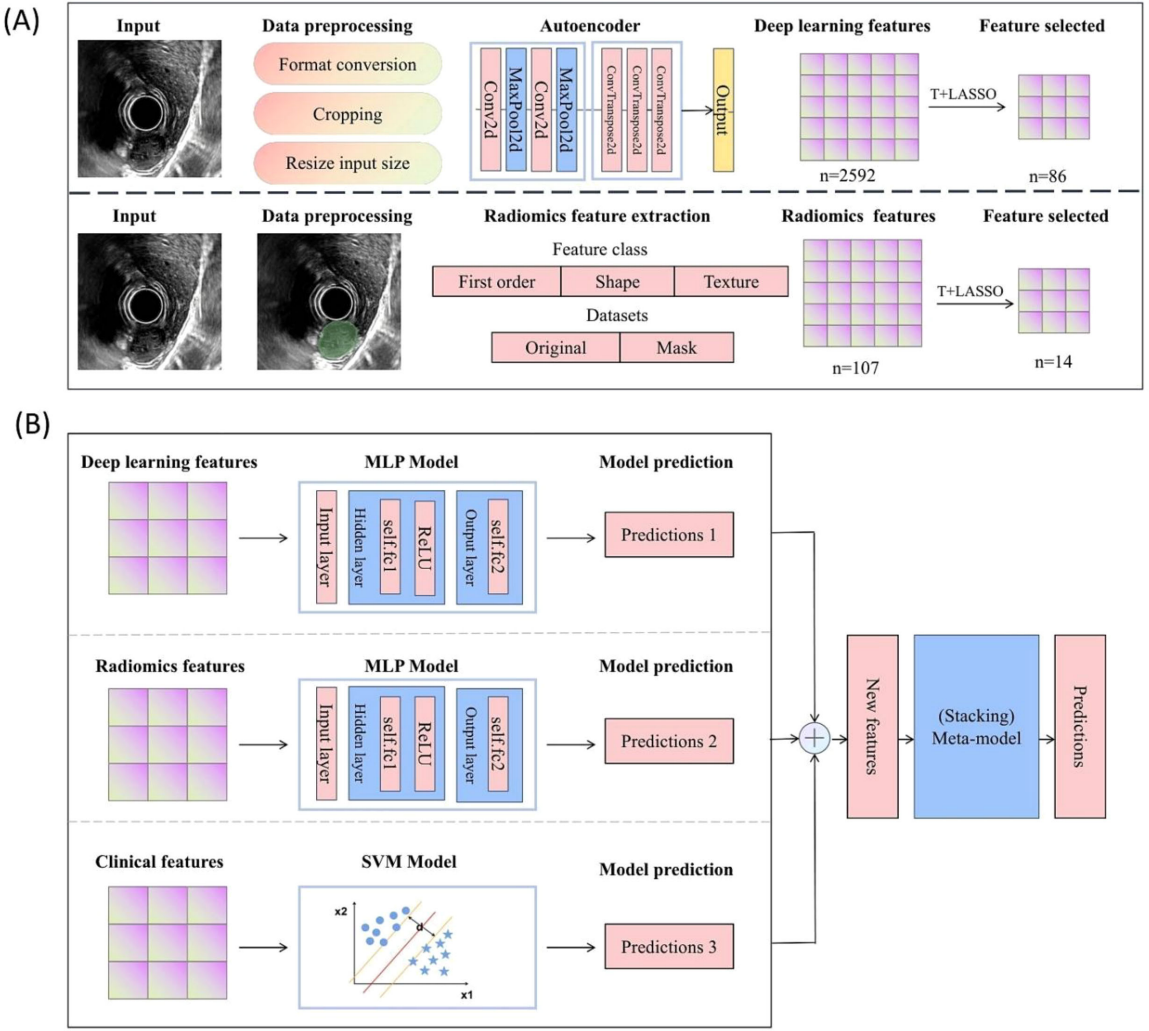
Method framework of the model **(A)** Diagram of the feature extraction phase **(B)** Diagram of the classification phase.

The proposed method consists of two main phases: feature extraction and classification. The flow diagram of the proposed method is shown in **Figures 1A, B**. In the feature extraction phase, multiple features from each image in the dataset are first extracted and pooled into the feature database, followed by feature selection. In the classification phase, multiple features are pooled into the classification models. Subsequently, a meta-model merges the results of these classification models, leading to the final predicted results. The following sections will provide detailed descriptions of the proposed method.

### 3.2 Equations

All collected images underwent a homogenization process that included the following steps: (1) data washing (removing patient information), (2) data denoising (eliminating unnecessary elements such as borders, text, and markers), (3) data standardization (ensuring uniform width and height for all images), and (4) data normalization. Radiomics models were utilized in this study, and the tumor regions were manually outlined by two radiologists using 3D Slicer software (open-source, version 5.2.2, http://www.slicer.org). The radiologists were blinded to the pathological results.

### 3.3 Deep learning feature extraction

**Figure 2:**
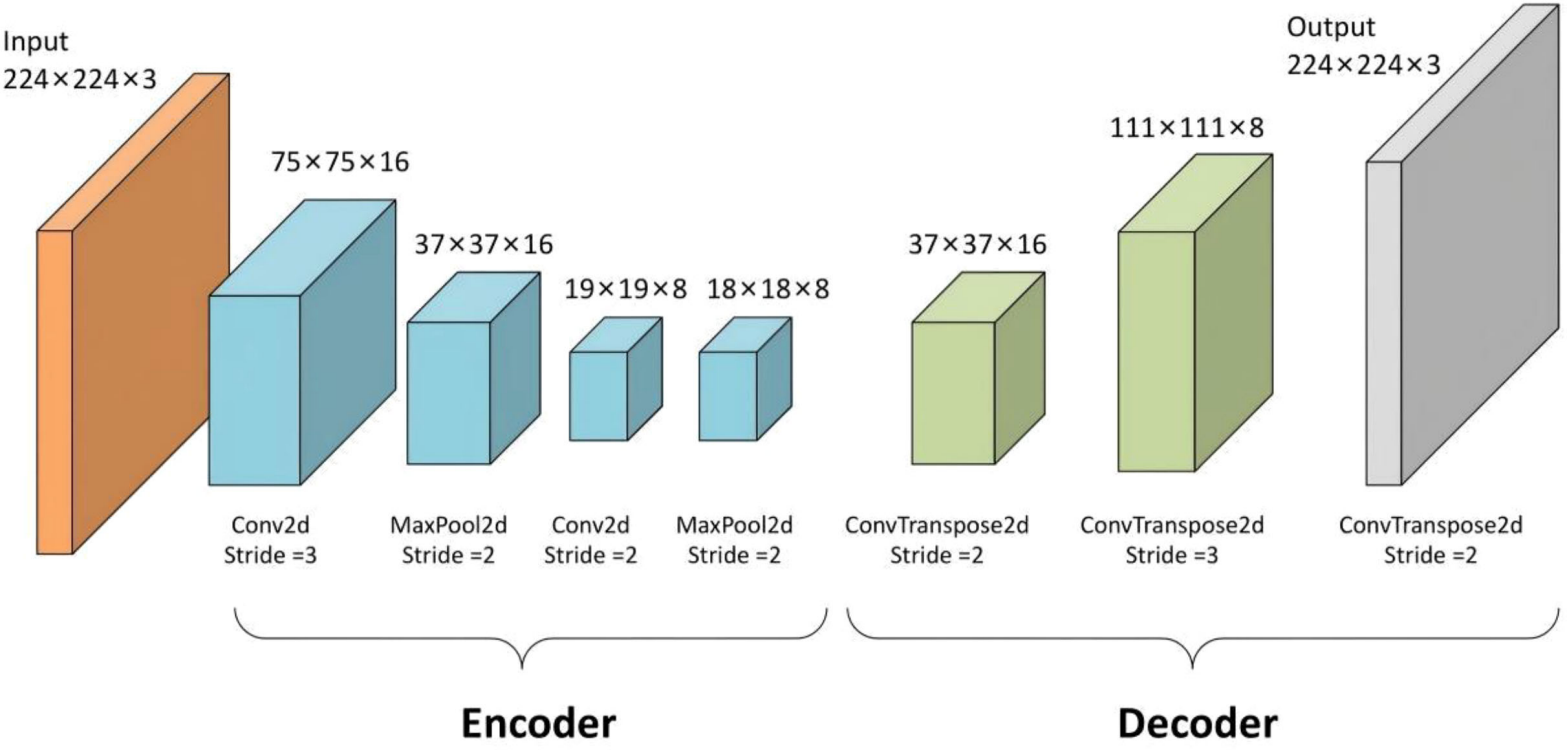
Autoencoder network architecture.

In this study, a CNN-based autoencoder model is used to extract deep learning features. The model consists of an encoder network and a decoder network. The encoder network includes four convolutional layers, while the decoder network has two layers. The structure of the network is illustrated in **Figure 2**.

In the encoder network, each encoder uses convolution with a filter bank to produce a series of feature maps. Specifically, the four convolutional layers have 32, 64, 128, and 256 filters, respectively. Each filter has a kernel size of 3×3 and a stride of 1. After each convolutional layer, batch normalization is applied to speed up training and stabilize the gradient flow. A ReLU activation function is then used to introduce non-linearities and improve the model’s representational capacity. Following this, max pooling is performed using a 2×2 window with a stride of 2 to reduce the number of parameters and extract key features. The final output of the encoder is a low-dimensional latent representation with a size of 14×14×256, which is then flattened into a 50,176-dimensional feature vector. This bottleneck structure ensures that the model retains only the most diagnostically relevant information while discarding noise and irrelevant variations.

The decoder network is designed to upsample the low-dimensional feature maps produced by the encoder in order to reconstruct the output, restoring it to the original input size. The decoder comprises two transposed convolutional layers, featuring 128 and 64 filters, respectively. Each layer is followed by batch normalization and a ReLU activation function. These feature maps are then deconvolved using a set of decoder filters to create dense feature maps. Following this process, the size of the feature map is resized to 224×224×3, and the pixel values are mapped to the interval of [-1, 1] using the Tanh activation function, resulting in the final output. The reconstruction loss is calculated as the mean squared error between the input images and the reconstructed images.

We chose a CNN-based autoencoder architecture for deep feature extraction for three main reasons. First, autoencoders allow for unsupervised pre-training, which helps the model learn meaningful feature representations from all available endoscopic ultrasound (EUS) images before we fine-tune it for the classification task. This is especially beneficial in medical imaging, where labeled data is often limited. Second, the bottleneck structure of the autoencoder naturally performs dimensionality reduction and denoising. This design compels the encoder to focus on capturing the most important features of gastric tumors while minimizing the impact of irrelevant signal variations. Third, the hierarchical feature learning capabilities of CNNs within the autoencoder are well-suited for capturing both intricate texture details and broader morphological features, as demonstrated by our SHAP analysis.

After the unsupervised pre-training phase, we discard the decoder and use the encoder network as a feature extractor. For each input EUS image, we extract the activations from the bottleneck layer. To reduce dimensionality and mitigate overfitting, we apply mean-pooling to these extracted deep learning features. The resulting low-dimensional feature representations are then prepared for subsequent classification tasks. We use the PyTorch package (http://www.pytorch.org) to extract the deep learning features.

### 3.4 Radiomic feature extraction

Radiomic features are extracted from the segmented gastric tumor using the pyradiomics package (version 3.0.1, https://www.pyradiomics.readthedocs.io/en/latest/). These features are categorized into three groups: geometry, intensity, and texture. Geometric features represent the two-dimensional shape characteristics of gastric tumors. Intensity features illustrate the statistical distribution of image intensities within the tumors. Texture features describe the patterns of spatial distributions of image intensities.

### 3.5 Feature selection

Feature selection is the process of identifying and retaining the most representative features while eliminating redundant ones. It can enhance model performance and prevent overfitting. In this context, t-tests and LASSO are used for the selection of deep learning features and radiomic features. Initially, t-tests are conducted to evaluate the correlation between each feature and the target variable. Subsequently, LASSO is employed to refine the selection based on the results from the t-tests. In the method, LASSO is performed by using the scikit-learn package (https://scikit-learn.org).

### 3.6 Model construction

A gastric tumor classification model was constructed by integrating deep learning features, radiomic features, and clinical features. The model combines the predictions of the three base models at the decision level. To clarify the construction process of each base model, the implementation steps for the deep learning feature model, the radiomic feature model, and the clinical feature model are detailed as follows.

To classify the selected deep learning features, a neural network model based on the Multilayer Perceptron architecture was used. The model consisted of an input layer, a hidden layer, and an output layer. The size of the input layer matched the dimensionality of the features, while the output layer contained 2 nodes, corresponding to the probabilities of the two categories. The hidden layer consisted of 32 nodes. The model was implemented using the PyTorch framework, utilizing CrossEntropyLoss as the loss function and the Adam optimizer for parameter updates. The training process lasted for 50 epochs.

The radiomic features were classified using a neural network model based on a Multi-Layer Perceptron. The size of the input layer matched the number of features selected after the feature selection process. Both the hidden layer, which consisted of 32 units, and the output layer, containing 2 units, were consistent with those used in the deep feature model. The training procedure adhered to the same protocol, utilizing PyTorch, CrossEntropyLoss, and the Adam optimizer. Hyperparameters were fine-tuned through cross-validation to enhance performance over 50 epochs.

The clinical feature model was built based on the support vector machine method and trained with ten-fold cross-validation. The effectiveness of the model was evaluated based on the AUC value.

### 3.7 Logistic regression-based model fusion method

To improve the predictive performance of gastric cancer classification, we employed logistic regression as a meta-model. This approach integrates the prediction results from our three base models: the deep learning feature model, the radiomics feature model, and the clinical feature model. By using the prediction probabilities from these models as input features, the logistic regression model learns linear weights and a bias term to generate a final classification probability, thus achieving an optimized data-driven combination. We applied L2 regularization to prevent overfitting, utilized the liblinear solver for parameter optimization, and set a fixed random seed to ensure reproducibility of the results. Recognizing the significance of selecting an appropriate threshold in clinical applications, particularly due to the class imbalance in our dataset (with GIST: non-GIST approximately at 1:4), we identified the optimal classification threshold using Youden’s index based on predictions from the training set. We evaluated thresholds within the range of [0.1, 0.9] in increments of 0.1. The threshold that maximized Youden’s index (J = sensitivity + specificity – 1) was selected as the final classification threshold, which was then applied to the test set for evaluation. This optimization strategy focuses on achieving a clinically relevant balance between sensitivity and specificity rather than solely optimizing for overall accuracy, which can be misleading in imbalanced datasets. By adaptively learning the contribution weights of each model, our method effectively captures the complementary information among the models, providing greater flexibility and superior predictive performance compared to fixed-weight fusion strategies.

### 3.8 Ablation study methodology

To thoroughly evaluate the contribution of each feature modality in our fusion framework, we designed a controlled ablation experiment. We constructed three dual-modality models by systematically excluding one type of feature from the complete set: (1) excluding clinical features (resulting in a model that relies only on Radiomics and Deep Learning), (2) excluding radiomics features (which uses only Clinical and Deep Learning), and (3) excluding deep learning features (leading to a model based on Clinical and Radiomics alone). All ablation models were built using the same fusion architectures and training protocols as the full model, with the only adjustments made to the input feature dimensions accordingly. We evaluated performance on an independent test set of 44 patients, utilizing standard metrics including specificity, sensitivity, accuracy, and AUC. The primary goal of this experiment was to assess the impact of removing each feature type on the model’s diagnostic performance, particularly focusing on the balance between sensitivity and specificity.

### 3.9 Statistical analysis

The statistical analysis was performed using Python (version 3.9), and the machine learning models were implemented using the scikit-learn package (http://www.scikit-learn.org/). T-tests and LASSO methods were employed to evaluate the deep learning features and radiomic features. The classification performance of the models, including specificity, sensitivity, positive predictive value (PPV), negative predictive value (NPV), accuracy (ACC), and AUC, was evaluated for both the training set and testing set using standard definitions.

## 4 Experiments

### 4.1 Experiment configuration

#### 4.1.1 Datasets

**Figure 3:**
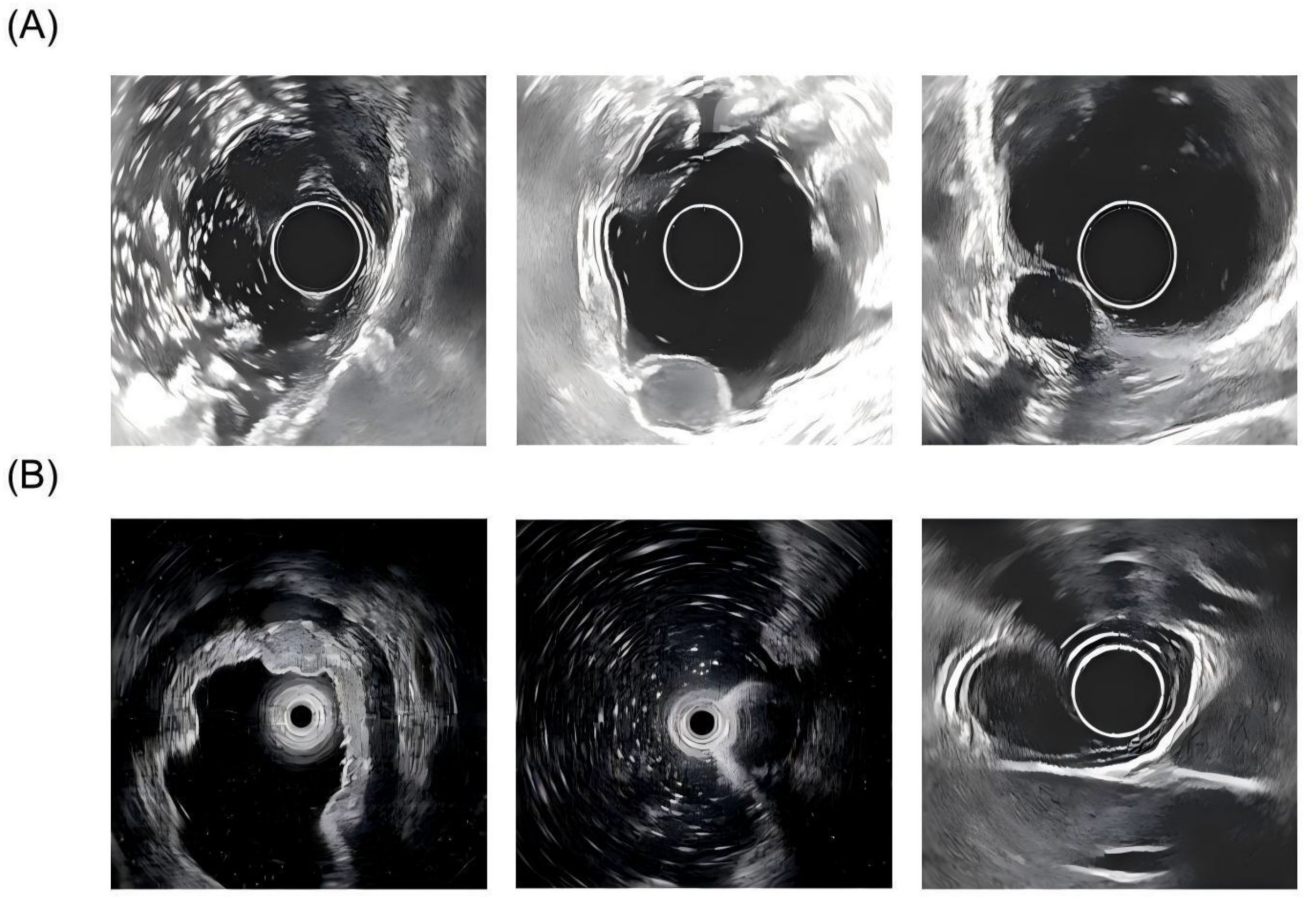
Representative endoscopic ultrasound images from the dataset. **(A)** Gastrointestinal stromal tumor. **(B)** Non-GIST lesions.

This study was approved by the Institutional Review Board (IRB) of the Affiliated Hospital of Chengde Medical University. The data were obtained from the Affiliated Hospital of Chengde Medical University and comprised ultrasound endoscopic images from 219 patients. In this group of patients, there were 43 cases of GIST (19.6%) and 176 cases of non-GIST lesions (80.4%), highlighting the relative rarity of GIST in clinical practice. The image dataset consisted of a total of 1,001 GIST images and 3,805 non-GIST images, summing up to 4,806 images overall. Informed consent was obtained from all participants, and the study was conducted in accordance with guidelines approved by the IRB. Representative samples are illustrated in **Figure 3**, where **Figure 3A** shows endoscopic ultrasound images of GIST, and **Figure 3B** presents images of non-GIST lesions, such as leiomyomas and schwannomas. Additionally, the dataset contains clinical information related to gastric tumors, including gender, age, tumor size, and tumor location.

To prevent data leakage and ensure a thorough evaluation, the dataset was partitioned strictly at the patient level. A total of 219 patients were randomly assigned to a training set and an independent testing set, consisting of 175 and 44 patients, respectively. Importantly, all images from any given patient were allocated exclusively to either the training set or the testing set, ensuring no overlap between the two groups. The training set includes images from 34 GIST patients and 141 non-GIST patients, while the testing set comprises images from 9 GIST patients and 35 non-GIST patients. Clinical data for these patients are presented in **Table 1**.

**Table 1:** Detailed distribution of the gastric tumor ultrasound endoscopy dataset in the training and testing sets.

Characteristic	Whole set (n=219)	Training set (n=175)	Testing set (n=44)	P-value
Sex^*^				0.631
Male	124(56.6%)	101(57.7%)	23(52.3%)	
Female	95(43.4%)	74(42.3%)	21(47.7%)	
Age^‡^ (mean ± SD)	60.3 ± 35.3	60.9 ± 39.3	57.9 ± 9.3	0.360
Size^‡^ (mean ± SD)	3.8 ± 8.9	3.9 ± 9.8	3.4 ± 3.8	0.645
Location^*^				0.591
Cardia	21(9.6%)	14(8.0%)	7(15.9%)	
Abdominal Cavity	1(0.5%)	1(0.6%)		
Esophagus	1(0.5%)	1(0.6%)		
Gastric Antrum	63(28.8%)	54(30.9%)	9(20.5%)	
Body-Fundus Junction	3(1.4%)	2(1.1%)	1(2.3%)	
Body-Antrum Junction	6(2.7%)	6(3.4%)		
Gastric Fundus	49(22.4%)	40(22.9%)	9(20.5%)	
Gastric Angle	20(9.1%)	16(9.1%)	4(9.1%)	
Gastric Body	54(24.7%)	40(22.9%)	14(31.8%)	
Pylorus	1(0.5%)	1(0.6%)		
Histopathology^*^				
GIST	43(19.6%)	34(19.4%)	9(20.5%)	1.000
Non-GIST	176(80.4%)	141(80.6%)	35(79.5%)	

^*^: Pearson’s chi-square test; ‡: T-tests; SD, Standard deviation; Means and standard deviations are retained to one decimal place, percentages to one decimal place, and p-values to three decimal places.

#### 4.1.2 Evaluation metrics

For the experiments, we selected four evaluation metrics: ACC, sensitivity, specificity, and AUC. The metrics are formally defined in Equations 1–3. These metrics are calculated using the following terms: True Positive (TP), True Negative (TN), False Positive (FP), and False Negative (FN). TP refers to cases that are correctly classified as positive when they are indeed positive. TN indicates cases that are correctly classified as negative when they are actually negative. FP represents cases that are incorrectly classified as positive, although they are negative. FN denotes cases that are incorrectly classified as negative when they are actually positive. In summary, FP and FN are the errors resulting from misclassification.

(1) $$\text{Accuracy}=\frac{\text{TP}+\text{TN}}{\text{TP}+\text{TN}+\text{FP}+\text{FN}}\times 100\%$$

(2) $$\text{Sensitivity}=\frac{\text{TP}}{\text{TP}+\text{FN}}\times 100\%$$

(3) $$\text{Specificity}=\frac{\text{TN}}{\text{FP}+\text{TN}}\times 100\%$$

### 4.2 Comparison with the state-of-the-art algorithms

#### 4.2.1 Comparison with EUS image classification methods

We conducted a comprehensive evaluation to compare the proposed multimodal fusion model with existing deep learning methods for EUS image classification.

First, as a baseline, we implemented the deep learning system introduced by Li et al. (44). Their method utilizes a ResNet50-based convolutional neural network, originally designed for identifying the originating mural layer of upper gastrointestinal submucosal tumors. We adapted this system for our binary classification task of distinguishing GIST from non-GIST lesions. When evaluated on our clinical dataset, this adapted baseline model achieved an accuracy of 0.86 and an AUC of 0.92, demonstrating its strong diagnostic capability as a benchmark.

In comparison, our proposed integrated model demonstrated superior discriminative ability, achieving an AUC of 0.95 compared to the baseline’s AUC of 0.92. While the overall accuracy of both models was comparable, their diagnostic profiles were strategically different. The baseline model exhibited a profile of high specificity (0.90) and moderate sensitivity (0.71). In contrast, our model achieved perfect sensitivity of 1.00, a critical attribute for minimizing missed diagnoses in a clinical screening context, though with a moderately lower specificity of 0.83. Furthermore, to contextualize our model’s performance against a contemporary approach for a related EUS classification task, we implemented the model architecture from Yi et al. (45) on our dataset. As detailed in **Table 2**, this implementation achieved an AUC of 0.74, an accuracy of 0.77, a sensitivity of 0.50, and a specificity of 0.85. Our multimodal fusion model exhibited consistently superior performance across all these metrics.

**Table 2:** Performance comparison of different deep learning models on endoscopic ultrasound image classification. Bold values indicate the best performance for each metric across all compared models.

Model	AUC	ACC	Sensitivity	Specificity
Li et al. (44)	0.92	0.86	0.71	**0.90**
Yi et al. (45)	0.74	0.77	0.50	0.85
**Ours**	**0.95**	**0.86**	**1.00**	0.83

Bold values indicate the best performance for each metric across all compared models.

Detailed comparative results are presented in **Table 2**.

#### 4.2.2 Comparison with public pre-trained CNNS

To evaluate the efficacy of the proposed multi-modal fusion approach, we performed comparative experiments with several widely-used CNN architectures pre-trained on ImageNet, including VGG-16, ResNet-50, ResNet-18, AlexNet, SqueezeNet, ShuffleNet, and DarkNet19 (46–53). Transfer learning was employed to leverage features learned from these pre-trained models for the new task. All models were fine-tuned end-to-end on our EUS image dataset under consistent experimental settings and hyperparameters to ensure a fair comparison.

As summarized in **Table 3**, the pre-trained models displayed varying performance profiles. VGG-16 achieved a relatively high AUC of 0.93 and a specificity of 0.90, though its sensitivity was limited to 0.69, reflecting a conservative predictive tendency that could lead to missed positive cases. While ResNet-50 delivered the highest specificity (0.92) among all models, it simultaneously recorded the lowest sensitivity (0.63), reflecting a performance trade-off that prioritizes ruling out negatives. Its AUC of 0.81 remained at a competitive level. ResNet-18 delivered notable accuracy of 0.87 and sensitivity of 0.87, albeit with moderately lower specificity of 0.85. In contrast, AlexNet’s results (with an AUC of 0.72 and an accuracy of 0.79) were consistently lower than those of more recent architectures. This indicates that while foundational, its representational capacity may be limited when applied to complex modern tasks such as gastric tumor classification from EUS images. Among lightweight models, SqueezeNet exhibited balanced performance with an AUC of 0.89 and an accuracy of 0.85, while demonstrating complementary strengths, ShuffleNet and DarkNet19 achieved specificities of 0.88 and sensitivities of 0.82, respectively.

**Table 3:** Performance comparison of different CNN models. Bold values indicate the best performance for each metric across all compared models.

Model	AUC	ACC	Sensitivity	Specificity
VGG-16 (47)	0.93	0.86	0.69	0.90
ResNet-50 (48)	0.81	0.86	0.63	**0.92**
ResNet-18 (49)	0.92	**0.87**	0.87	0.85
AlexNet (50)	0.72	0.79	0.72	0.67
SqueezeNet (51)	0.89	0.85	0.85	0.78
ShuffNet (52)	0.88	0.84	0.76	0.88
DarkNet19 (53)	0.87	0.83	0.82	0.83
**Ours**	**0.95**	0.86	**1.00**	0.83

Bold values indicate the best performance for each metric across all compared models.

The proposed multi-modal fusion model achieved outstanding performance across key metrics, an AUC of 0.95, and a sensitivity of 1.00, surpassing the compared models. This advantage is particularly crucial in clinical diagnostics, as the exceptionally high sensitivity demonstrated by the model indicates its capability to identify target patients with remarkable effectiveness, which is crucial for ensuring that high-risk cases are not missed and that timely clinical intervention can be initiated.

In conclusion, the proposed multi-modal fusion framework integrates deep features, radiomic features, and clinical parameters, leading to more comprehensive and robust performance in gastric tumor classification tasks compared to single-modality pre-trained models.

#### 4.2.3 Comparison with machine learning methods

To thoroughly evaluate the performance of the proposed multimodal fusion framework, this study compared it with four widely-used traditional machine learning algorithms: Support Vector Machine (SVM) (54), Random Forest (RF) (55), eXtreme Gradient Boosting (XGBoost) (56), and Logistic Regression (LR) (57). All comparative experiments were conducted using the same preprocessed feature set to ensure a fair comparison.

The experiments utilized 104-dimensional feature vectors derived from multimodal data, consisting of 86 deep learning features, 14 radiomics features, and 4 clinical features. The dataset was randomly split into training and testing sets with an 80:20 ratio using stratified sampling to preserve the original class distribution. All continuous features were standardized to reduce the influence of feature scale variations on model performance.

Performance metrics for the baseline models are summarized in **Table 4**. Compared to these methods, the proposed integrated learning framework exhibited improvements across key performance indicators. In particular, the proposed method achieved higher sensitivity while maintaining competitive levels of accuracy and AUC. The increase in sensitivity indicates a strengthened capability to identify positive cases, addressing a common limitation of traditional machine learning models in medical image classification tasks. This aspect may be particularly relevant for applications such as early disease detection and clinical decision support.

**Table 4:** Performance comparison of baseline models with multimodal feature fusion. Bold values indicate the best performance for each metric across all compared models.

Model	AUC	ACC	Sensitivity	Specificity
SVM (54)	0.91	0.85	0.55	**0.93**
LR (57)	0.91	0.87	0.63	**0.93**
RF (55)	0.93	0.84	0.50	**0.93**
XGBoost (56)	0.94	**0.88**	0.72	0.67
**Ours**	**0.95**	0.86	**1.00**	0.83

Bold values indicate the best performance for each metric across all compared models.

#### 4.2.4 Comparison with contemporary fusion methods

To thoroughly assess the performance of the proposed decision-level fusion model and to position it within the current research context of multimodal medical image analysis, this study conducted a systematic comparison with three fusion methods documented in recent literature. All comparative experiments used the same EUS gastric tumor dataset with identical training and testing splits to ensure a fair evaluation. The dataset consisted of 4,806 EUS images from 219 patients, featuring multimodal attributes including deep learning features, radiomics features, and clinical features of the patients. To mitigate scale effects, all continuous features were standardized to have a mean of zero and a variance of one.

The performance metrics of contemporary fusion methods are summarized in **Table 5**. The feature-level fusion method proposed by Lan et al. (58) demonstrated strong performance on our dataset, achieving an ACC of 0.84. In contrast, the weighted fusion strategy employed by Nijiati et al. (59) achieved a notably higher AUC of 0.95, along with improved sensitivity of 0.78 and specificity of 0.91. Our proposed model also reached a high AUC of 0.95 while achieving a perfect sensitivity of 1.00, indicating an exceptional capability to identify all positive cases. However, this came with a trade-off in specificity, which was measured at 0.83 compared to the other methods.

**Table 5:** Performance comparison of contemporary multimodal fusion models. Bold values indicate the best performance for each metric across all compared models.

Model	AUC	ACC	Sensitivity	Specificity
Lan et al. (58)	0.81	0.84	0.67	0.89
Nijiati et al. (59)	**0.95**	**0.89**	0.78	**0.91**
**Ours**	**0.95**	0.86	**1.00**	0.83

Bold values indicate the best performance for each metric across all compared models.

Compared to contemporary methods, the proposed decision-level fusion framework demonstrated superior overall performance, achieving the highest AUC of 0.95 among all models tested. Most notably, our method achieved a perfect sensitivity of 1.00, ensuring that all positive cases were reliably identified. This advantage is crucial in clinical diagnostics, where failing to detect a true positive can carry significant risks. While there was a slight trade-off in specificity at 0.83, this balance indicates that the proposed approach successfully prioritizes the detection of true cases without significantly compromising the ability to rule out negatives. This effectively addresses a key challenge in real-world diagnostic scenarios, where both overdiagnosis and underdiagnosis need to be carefully managed.

The superior performance of our decision-level fusion model can be attributed to several methodological advantages. First, the modular design allows each sub-model to be optimized independently with algorithms that are best suited to its specific feature types. This approach avoids the potential dilution of information that can occur in early fusion methods. Second, the ensemble mechanism offers robustness against noise or misleading features from any single modality. In contrast, feature-level fusion methods may propagate errors through combined feature vectors. Finally, the explicit separation of different feature categories enhances the model’s interpretability, enabling clinicians to understand how imaging and clinical information contribute to the final diagnosis.

#### 4.2.5 Comparison with state-of-the-art deep learning architectures

To benchmark our autoencoder-based feature extraction against more recent deep learning architectures, we conducted additional experiments comparing it with EfficientNet (B0, B3, B7) and the Vision Transformer. EfficientNet is a family of convolutional neural network (CNN) architectures that achieves strong performance through the compound scaling of depth, width, and resolution. The Vision Transformer, on the other hand, applies a transformer architecture originally designed for natural language processing to image classification by dividing an image into patches and processing them as sequences. All models were trained and evaluated under identical experimental conditions to ensure a fair comparison. Specifically, we maintained the same training, validation, and test splits, along with consistent data augmentation strategies, optimizer settings (Adam), a learning rate of 0.001, a batch size of 32, and 100 epochs across all architectures. For the EfficientNet models, we used pre-trained weights on ImageNet and fine-tuned them on our EUS dataset. For the Vision Transformer (ViT-S), we used a standard patch size of 16×16 and also initialized it with ImageNet pre-trained weights. The results of these experiments are summarized in **Table 6**.

**Table 6:** Performance comparison of different deep learning architectures. Bold values indicate the best performance for each metric across all compared models.

Model	AUC	ACC	Sensitivity	Specificity
EfficientNet-B0 (60)	0.79	0.84	0.78	0.86
EfficientNet-B3 (61)	0.82	0.86	0.78	0.89
EfficientNet-B7 (62)	0.78	**0.89**	0.78	**0.91**
Vision Transformer (63)	0.83	0.84	0.56	**0.91**
**Ours**	**0.95**	0.86	**1.00**	0.83

Bold values indicate the best performance for each metric across all compared models.

Among the compared models, EfficientNet-B7 achieved the highest accuracy (0.89) and specificity (0.91), while EfficientNet-B3 achieved the highest AUC (0.82) among the EfficientNet family. The Vision Transformer achieved an AUC of 0.83 but showed lower sensitivity (0.56) compared to the EfficientNet models (0.78). Transformers typically require large-scale pre-training datasets to learn generalizable visual representations, whereas our dataset contains 219 patients, which may limit ViT training. In contrast, our autoencoder, with its CNN-based inductive biases and unsupervised pre-training capability, achieved an AUC of 0.95 and a sensitivity of 1.00 on the same test set.

#### 4.2.6 Comparison with alternative feature selection methods

To benchmark our t-test and LASSO feature selection approach, we compared its performance with two widely used alternative methods, i.e. mutual information (MI) and recursive feature elimination (RFE). Mutual information is a filter-based method that measures the non-linear dependency between each feature and the target variable. Recursive feature elimination is a wrapper method that recursively removes features with the smallest weights from a logistic regression model. To ensure a fair comparison, all three methods were evaluated using the same data split and the same classification threshold (0.2) as in the main analysis.

The results are summarized in **Table 7**. Our proposed method achieved the highest AUC (0.95) and accuracy (0.86) among the three methods, with a perfect sensitivity of 1.00. Mutual information attained a comparable AUC (0.90) and also achieved perfect sensitivity (1.00), but with slightly lower specificity (0.80 vs. 0.83) and accuracy (0.84 vs. 0.86). In contrast, recursive feature elimination exhibited substantially lower sensitivity (0.33), despite achieving the highest specificity (0.86), indicating that it struggled to correctly identify positive cases. Regarding feature selection efficiency, MI selected a more parsimonious set of features (20 deep learning features and 20 radiomics features) compared to our method (86 deep learning features and 14 radiomics features) and RFE (86 deep learning features and 14 radiomics features).

**Table 7:** Performance comparison of different feature selection methods. Bold values indicate the best performance for each metric across all compared models.

Method	AUC	ACC	Sensitivity	Specificity
Mutual Information (64)	0.90	0.84	**1.00**	0.80
Recursive Feature Elimination (65)	0.82	0.75	0.33	**0.86**
**Ours**	**0.95**	**0.86**	**1.00**	0.83

Bold values indicate the best performance for each metric across all compared models.

Overall, while mutual information demonstrated competitive performance with fewer features, our t-test combined with the LASSO method showed superior AUC and accuracy, which are crucial for the clinical task of gastric tumor classification. Given its balance between predictive performance and feature sparsity, along with the interpretability of LASSO coefficients for subsequent SHAP analysis, we chose the t-test and LASSO combination as our primary feature selection approach.

### 4.3 Analysis and discussion

In this section, we further analyze the performance of the proposed method, which includes the analysis of the proposed multi-modal framework. We also analyze the feature importance.

#### 4.3.1 Comparison with baseline single-modality models

Three single-modality models, namely deep learning, radiomics, and clinical feature-based approaches, were independently developed and evaluated to assess their respective capabilities in classifying gastric tumors using EUS images.

**Figure 4:**
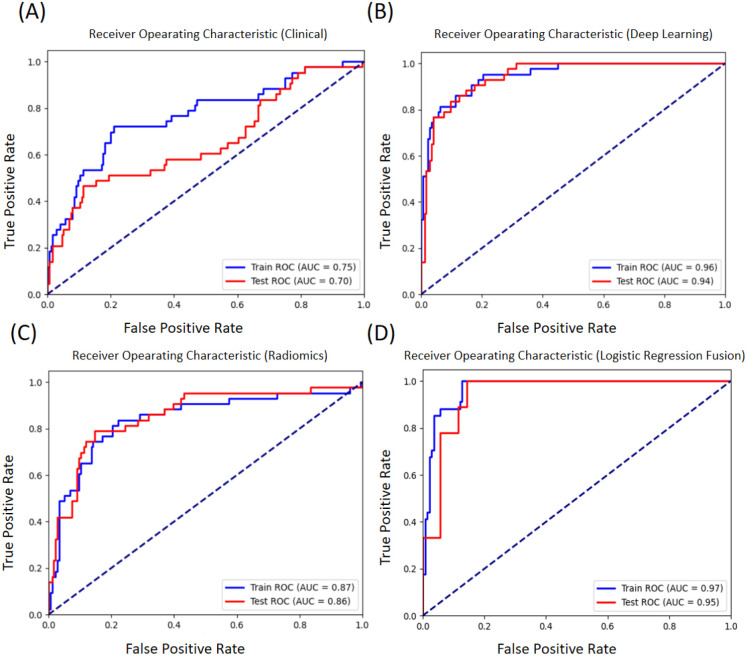
Average ROC curves in the training set and testing set **(A)** The ROC curve for the clinical feature model **(B)** The ROC curve for the deep learning feature model **(C)** The ROC curve for the radiomics feature model **(D)** The ROC curve for the full fusion model.

For the clinical feature model, a support vector machine (SVM) algorithm was used. Performance results displayed in **Figure 4A** indicate diagnostic utility. These findings underscore the constraints of using clinical parameters alone, while also establishing a baseline for multimodal integration. Furthermore, this model may offer complementary diagnostic value for cases with ambiguous imaging manifestations, thereby supporting clinical decision-making in specific scenarios.

In this study, an autoencoder was employed to extract deep learning features from EUS images, followed by classification using a multilayer perceptron. The unsupervised feature extraction process enabled the learning of latent image representations while preserving discriminative power and reducing dimensionality. As shown in **Figure 4B**, the model achieved an AUC of approximately 0.96 on the training set and 0.94 on the testing set. These results indicate the usefulness of autoencoder-derived features for gastric tumor classification. Compared to conventional handcrafted feature extraction methods, this approach exhibited enhanced representational capacity; however, performance remains limited for rare tumor subtypes with insufficient samples.

The radiomics model, whose results are presented in **Figure 4C**, attained mean AUC values of 0.87 on the training set and 0.86 on the test set. These outcomes affirm the relevance of radiomic features in gastric tumor classification, though the performance was moderately lower than that of the deep learning model. The small difference between training and testing performance suggests that the feature representation is generalizable and not overfit.

The full fusion model, which integrates deep learning, radiomics, and clinical features, demonstrates superior performance as shown in **Figure 4D**. This multimodal approach achieved significantly higher AUC values, reaching 0.97 on the training set and 0.95 on the testing set, outperforming all single-modality models. The enhanced performance validates the synergistic effect of combining complementary information from different data modalities, where each modality contributes unique diagnostic insights that collectively improve classification accuracy and robustness.

#### 4.3.2 Effectiveness of multi-modal fusion

**Figure 5:**
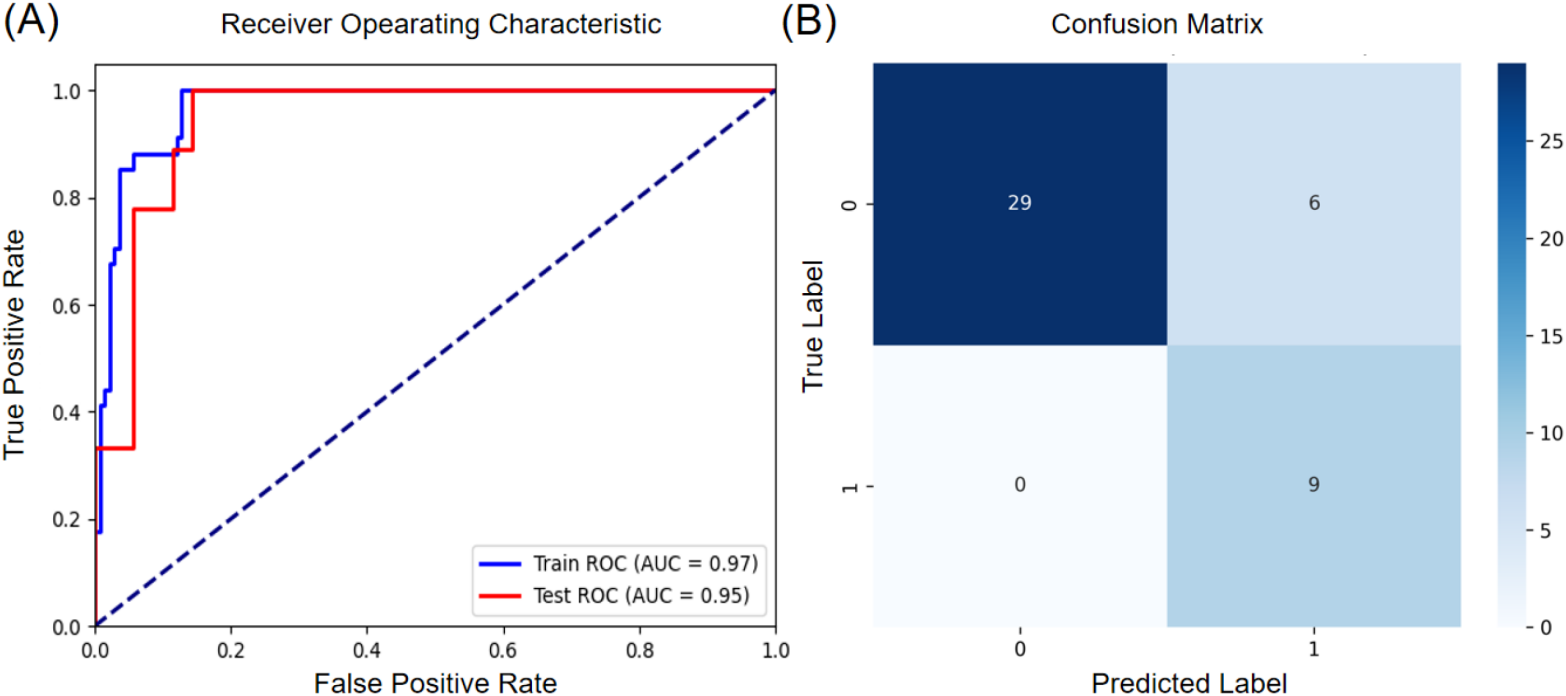
Model performance evaluation **(A)** ROC curve for meta-model **(B)** Confusion matrix of the model on the testing set.

In addition to the baseline models, the three individual models were integrated via a stacking ensemble learning approach, using logistic regression (LR) as the meta-learner. The corresponding ROC curves are shown in **Figure 5A**. Using an optimal threshold of 0.2, which was determined by maximizing Youden’s index on the training set to address class imbalance, the model achieved an AUC of 0.97 on the training set and 0.95 on the test set. The subsequent performance metrics, summarized in **Table 8** and illustrated in **Figure 5**, are evaluated at this optimized threshold. On the training set, the model exhibited a sensitivity of 100.00%, a specificity of 87.23%, and an accuracy of 89.71%. In the test set, these values remained at 100.00% for sensitivity, while specificity was 82.86% and accuracy was 86.36%. The confusion matrix for the test set is presented in **Figure 5B**.

**Table 8:** Diagnostic performance of the model in differentiating GISTs from non-GISTs.

Diagnosis	Sensitivity,%	Specificity,%	ACC,%	PPV,%	NPV,%
Training set	100.00 (89.85-100.00)	87.23 (81.73-92.74)	89.71 (85.21-94.21)	65.38 (52.45-78.32)	100.00 (96.97-100.00)
Testing set	100.00 (70.09-100.00)	82.86 (70.37-95.34)	86.36 (76.22-96.50)	60.00 (35.21-84.79)	100.00 (88.30-100.00)

Values in parentheses are 95% CIs. NPV, negative predictive value; PPV, positive predictive value; CI, confidence interval.

The results indicate that the stacking ensemble method leads to improved performance in gastric tumor classification. This improvement can be attributed to the method’s capacity to integrate complementary predictive information from diverse models, thereby facilitating a more comprehensive analysis (66).

The proposed multimodal fusion approach demonstrates distinct advantages in the classification of gastric tumors, as quantitatively and visually validated. By integrating 86 deep learning features, 14 radiomic features, and 4 clinical parameters (gender, age, tumor size, and tumor location), the fusion model, developed using ten-fold cross-validation, achieved AUC values of 0.97 on the training set and 0.95 on the testing set.

Compared to unimodal approaches, the fusion method demonstrated superior performance. It not only outperformed both the clinical model, which achieved a training AUC of 0.75 and a testing AUC of 0.70, and the radiomics model, which achieved a training AUC of 0.87 and a testing AUC of 0.86, but its final performance with a training AUC of 0.97 and a testing AUC of 0.95, also slightly exceeded that of the best-performing standalone deep learning model, which achieved a training AUC of 0.96 and a testing AUC of 0.94.

Furthermore, the fusion approach showed consistent robustness across diverse patient subgroups, with particularly strong performance gains observed in clinically challenging cases. The high sensitivity of 100% achieved in both sets, resulting from the optimized threshold strategy, is crucial for clinical screening since missing a GIST case poses a significant risk.

#### 4.3.3 Ablation study and comparative analysis

To validate the necessity of integrating all three feature modalities and to quantify the contribution of each component, we conducted a systematic ablation study comparing our full fusion model to three dual-modality variants. The results were shown in **Table 9**.

**Table 9:** Bold values indicate the best performance for each metric across all compared models.

Model	Features Included	Specificity,%	Sensitivity,%	ACC,%	AUC	P-value*
Full Fusion Model	Clinical + Radiomics + DL	**82.86**	**100.00**	**86.36**	**0.95**	–
Ablation Model A	Radiomics + DL	74.29	100.00	79.55	**0.95**	0.719
Ablation Model B	Clinical + DL	80.00	77.78	79.55	0.88	0.218
Ablation Model C	Clinical + Radiomics	71.43	100.00	77.27	0.90	0.078

^*^: P-values calculated using DeLong’s test comparing AUC against the full fusion model.

Bold values indicate the best performance for each metric across all compared models.

The comprehensive model, which incorporates Clinical data, Radiomics, and Deep Learning, achieved impressive overall performance, exhibiting a balanced sensitivity and specificity. It reached an accuracy of 86.36% and an AUC of 0.95. Additionally, the model demonstrated clinically significant performance with a sensitivity of 100.00% and a specificity of 82.86%, effectively distinguishing between GIST and non-GIST lesions.

Ablation Model A, which combines Radiomics and Deep Learning, demonstrated a sensitivity of 100% but had reduced specificity at 74.29%, resulting in an overall accuracy of 79.55%. This model achieved an AUC of 0.95, the same as the full fusion model. However, DeLong’s test showed no statistically significant difference between the two models (P = 0.719). This indicates that clinical features do not significantly enhance the AUC in this configuration, despite their contribution to improved specificity. Ablation Model B, which combines Clinical features with Deep Learning, showed relatively balanced performance with a sensitivity of 77.78% and a specificity of 80.00%, leading to an accuracy of 79.55% and an AUC of 0.88. A statistical comparison using DeLong’s test indicated that the full model had a numerically higher AUC than Ablation Model B, but this difference did not reach statistical significance (P = 0.218). Ablation Model C, which consists of Clinical features and Radiomics, achieved a sensitivity of 100% and a specificity of 71.43%. Its accuracy was 77.27%, and it had an AUC of 0.90. The absence of deep learning features resulted in reduced specificity compared to the full model. The difference in AUC between the full model and Ablation Model C approached but did not achieve statistical significance (P = 0.078), suggesting a trend toward the importance of deep learning features in maintaining both high sensitivity and specificity.

A formal comparison of AUC values using DeLong’s test for correlated ROC curves showed that while the full model had a numerically higher AUC compared to both Ablation Model B and Ablation Model C, these differences were not statistically significant at the conventional α = 0.05 level. Specifically, the p-values were as follows: vs. Model A: P = 0.719; vs. Model B: P = 0.218; vs. Model C: P = 0.078. This lack of statistical significance may be due to the limited sample size in the test set (n = 44), which reduces the statistical power to detect modest performance differences. However, the consistent trend across all comparisons supports the clinical relevance of the full fusion approach.

The results of the ablation study provide compelling evidence for several key points: (1) each feature modality contributes unique and complementary diagnostic information, even though the contributions to the Area Under the Curve (AUC) may not be statistically significant in smaller sample sizes; (2) excluding any single modality disrupts the optimal balance between sensitivity and specificity. Specifically, omitting clinical or radiomic features significantly affects specificity, while excluding deep learning features impacts the overall balance; (3) deep learning features are essential for achieving the best combination of high sensitivity and adequate specificity; (4) the synergistic integration of all three modalities results in optimal clinical performance, which was not fully achieved by any combination of two modalities in this study. This integration allows for achieving 100% sensitivity while maintaining 82.86% specificity.

#### 4.3.4 Feature Importance and Interpretability

##### 4.3.4.1 Visualize top discriminative radiomics features

**Figure 6:**
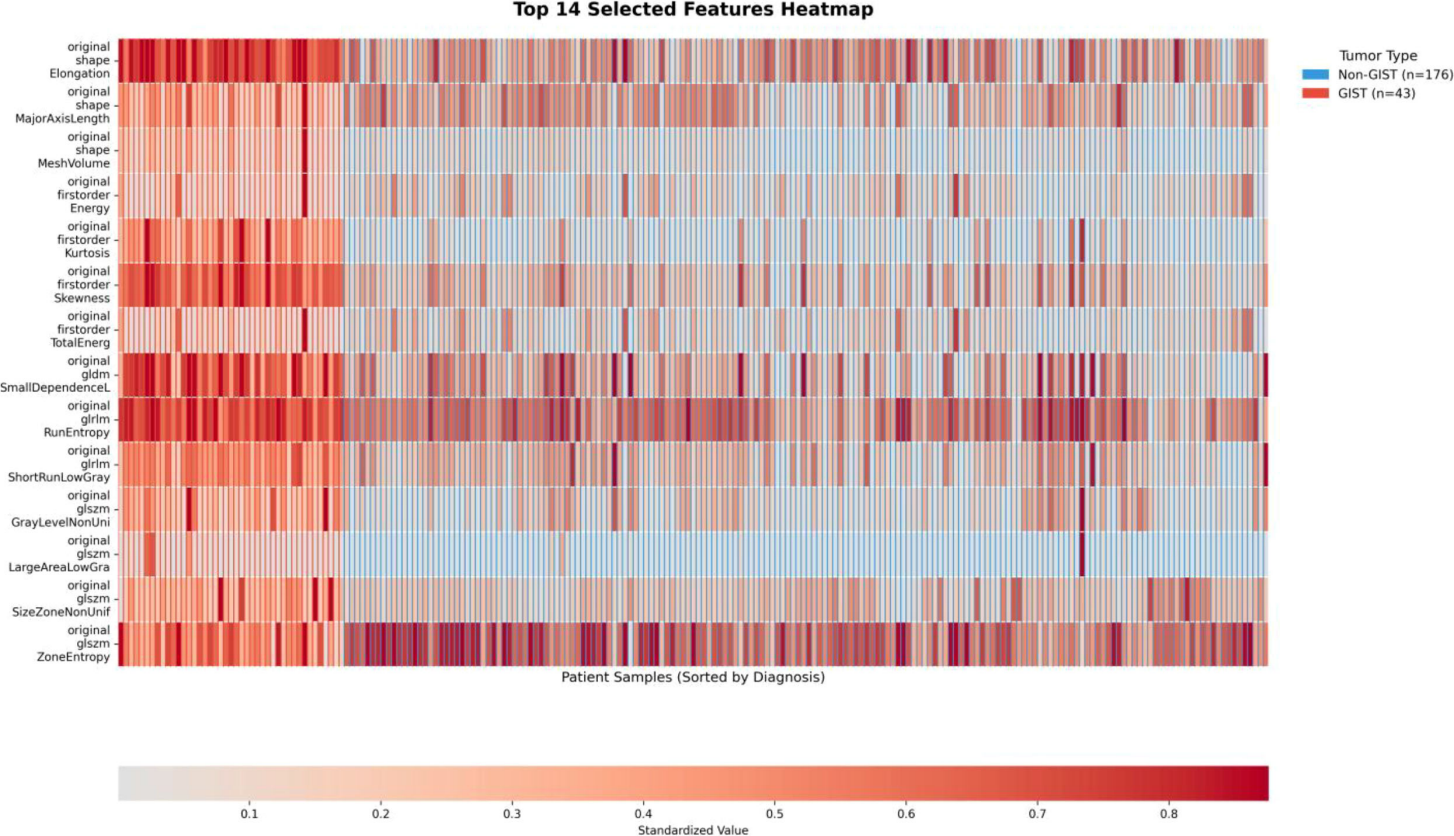
Heatmap analysis of radiomics features: visualization of heterogeneity patterns in GIST vs. non-GIST tumors.

This study employed a two-stage feature selection method to analyze radiomic features in gastric tumors. In the first stage, t-tests were performed (α is set as 0.05) on 107 original features, leading to the retention of 90 features that were statistically significant. Subsequently, LASSO regression with 10-fold cross-validation (random seed set to 42) was applied for further refinement, identifying 14 highly discriminative features. To visualize the distribution patterns of these features, a heatmap was generated, as shown in **Figure 6**, which displays standardized feature values grouped by diagnosis. Features were compared between GIST and non-GIST cases. Hierarchical clustering based on Euclidean distance was applied, with sample groups color-coded as follows: GIST samples in red and non-GIST samples in blue.

The analysis yielded several notable observations regarding the selected features. First-order statistical features demonstrated discriminative capacity, with original_firstorder_Skewness (with a coefficient of +0.126) and original_firstorder_Kurtosis (with a coefficient of +0.114) showing higher values in GIST cases. These patterns suggest asymmetric and peaked intensity distributions, which may reflect tumor heterogeneity (44). Morphological features such as original_shape_Elongation (with a coefficient of +0.096) and original_shape_MeshVolume (with a coefficient of +0.023) were positively correlated with GIST, indicating a tendency toward elongated shapes and larger volumes.

Texture analysis indicated that original_glszm_LargeAreaLowGrayLevelEmphasis (with a coefficient of –0.064) was negatively associated with GIST, implying the presence of fewer extensive hypodense areas. Original_glrlm_ShortRunLowGrayLevelEmphasis (with a coefficient of –0.030) suggested more complex textural patterns in GIST cases. The heatmap visualization, as shown in **Figure 6**, illustrates these distributional differences: GIST cases exhibit higher values (shown in red) for features such as kurtosis and skewness, whereas non-GIST cases show more prominent texture uniformity. Additionally, original_glszm_ZoneEntropy (with a coefficient of –0.019) exhibited an inverse correlation with GIST, a result that merits further investigation as it may indicate distinct textural homogeneity within this cohort.

##### 4.3.4.2 Visualize top discriminative deep learning features

**Figure 7:**
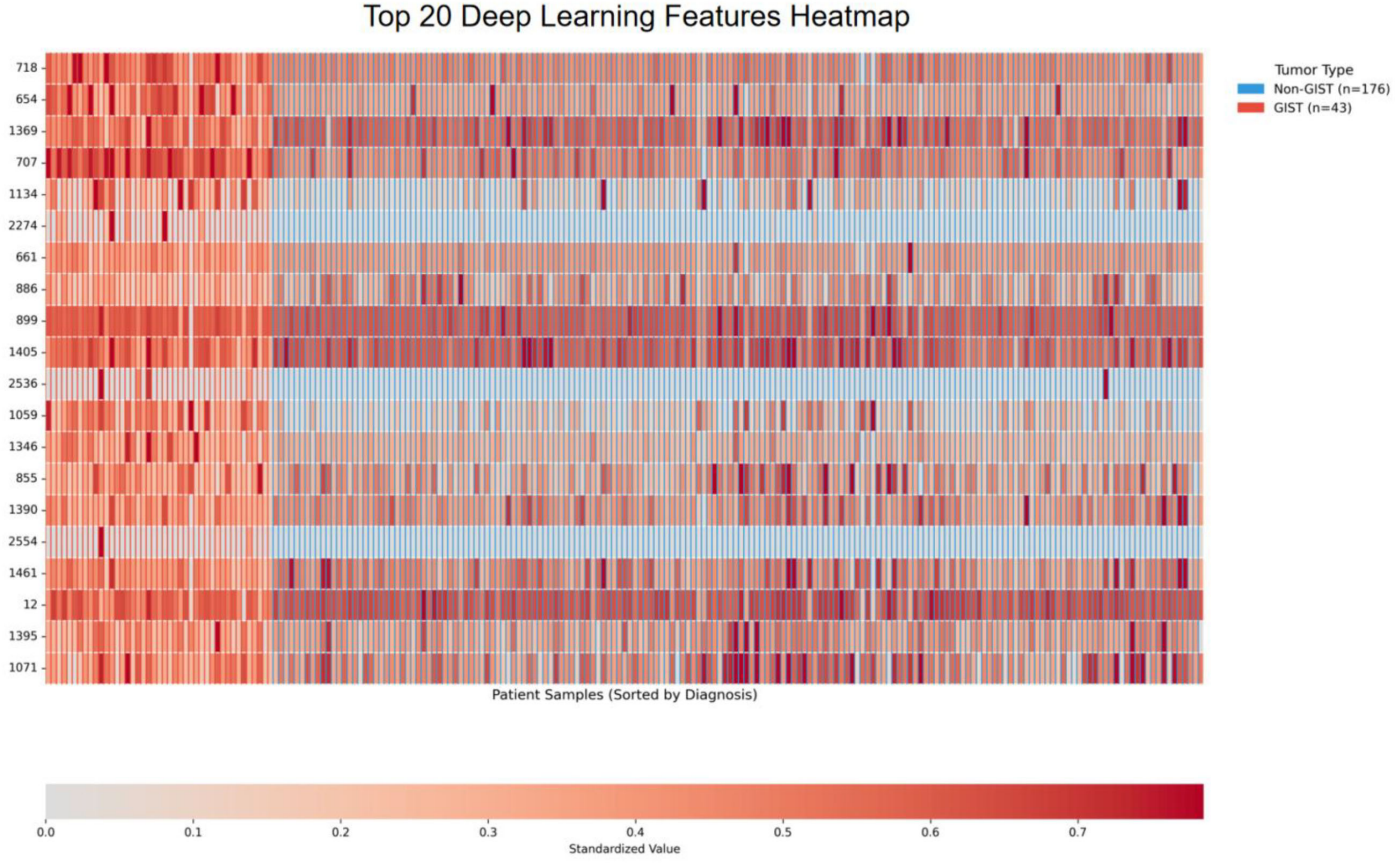
Heatmap analysis of DEEP learning features: visualization of heterogeneity patterns in GIST vs. non-GIST tumors.

**Figure 7** displays a heatmap illustrating the 20 most influential deep learning features selected based on their LASSO coefficient importance. This heatmap shows standardized expression patterns across 219 patient samples, which include 176 samples from non-GIST patients and 43 samples from GIST patients. The samples are arranged by diagnosis along the horizontal axis, with non-GIST samples on the left and GIST samples on the right. Meanwhile, the features are organized by their importance along the vertical axis, with the most discriminative features positioned at the top. The color coding ranges from light gray to deep red, representing standardized feature expression levels; a deeper red indicates higher expression in the corresponding samples.

The analysis of the selected features shows that Feature_718 has a coefficient of +0.100, making it the most positively correlated feature, while Feature_1369, with a coefficient of -0.050, is the most negatively correlated feature. Among the top 20 features, there is a balanced distribution: 11 features are positively associated with GIST, and 9 features are negatively associated. This results in an average absolute coefficient of 0.036. This pattern suggests a systematic differential expression of these deep learning features between the two tumor types.

The visualization reveals three key patterns: First, there is a noticeable clustering of feature expression, with high-importance features such as 718, 1369, and 707 showing significantly elevated expression (indicated by deep red) in GIST patients compared to their lower expression (shown as light orange/blue) in non-GIST patients. Second, there is a pronounced directional consistency in features such as 1134, 2274, and 2536, which consistently exhibit low expression (depicted in blue/light gray) in non-GIST patients. Third, the clear color differentiation between diagnostic groups along the sample axis highlights the combined classification efficacy of these 20 features, providing intuitive visual evidence for the interpretability of the model.

Although inherently abstract, deep learning features effectively capture complex hierarchical patterns and spatial relationships within tumor architecture using convolutional neural networks. The observed differences in expression patterns suggest that these features can identify subtle textural and structural characteristics that distinguish GIST from non-GIST tumors. This differentiation may reflect variations in cellular organization, vascular patterns, or stromal composition that traditional radiomics methods struggle to quantify. Additionally, the complementary nature of deep learning features alongside radiomic measurements offers a multi-faceted view of gastric tumor heterogeneity, enhancing both the robustness and clinical applicability of the diagnostic framework.

##### 4.3.4.3 Quantitative interpretation of radiomics features using SHAP

To understand the individual contribution of the 14 key radiomics features selected using LASSO, we conducted an attribution analysis based on Shapley Additive Explanations (SHAP) values. In this approach, we treat the model’s predictive output as a cooperative game involving all features. SHAP values fairly allocate the contribution of each feature to the outcome of the prediction for a given sample, allowing for consistent and comparable explanations at both global and local levels. Importantly, this analysis connects quantitative imaging biomarkers with clinical reasoning by identifying not only which features are important but also how their specific values influence the model’s decision regarding a particular diagnosis. This layer of interpretability is essential for transforming model outputs into clinically actionable insights and for building trust with medical practitioners.

The quantitative contribution metrics for all selected features are summarized in **Table 10**. The SHAP analysis revealed that the contributions of radiomics features to model predictions exhibited a distinct hierarchical structure and varying directions of influence. Among the morphological features, the original_shape_Elongation had the most significant contribution, with a SHAP value range of 0.308. High values (indicated by red point clusters in the figure) were primarily concentrated in the positive region (0.10–0.20), significantly influencing predictions towards GIST. This finding aligns with established CT imaging literature: GISTs typically exhibit an exophytic or dumbbell-shaped growth pattern, often protruding outward from the gastric wall, which results in an elongated tumor morphology on axial images (67). In contrast, gastric adenocarcinomas tend to demonstrate infiltrative growth along the gastric wall with ill-defined margins, contributing to less elongated or more irregular shapes (68). Therefore, an elongated tumor shape may serve as a key imaging marker indicating GIST rather than gastric cancer.

**Table 10:** Descriptive statistics of SHAP values for radiomics features.

Feature	Min	Max	Range	Mean	Std
original_shape_Elongation	-0.076554	0.231488	0.308042	-0.001313	0.069295
original_firstorder_Kurtosis	-0.081113	0.157696	0.238808	-0.001174	0.080856
original_firstorder_Skewness	-0.070551	0.128944	0.199495	-0.005806	0.064431
original_shape_MeshVolume	-0.012482	0.110522	0.123005	0.00022	0.013971
original_firstorder_TotalEnergy	-0.023748	0.071744	0.095492	-0.000145	0.022277
original_glszm_LargeAreaLowGrayLevelEmphasis	-0.032752	0.054993	0.087745	-0.001102	0.019727
original_firstorder_Energy	-0.017654	0.06068	0.078334	0.000729	0.017774
original_glrlm_RunEntropy	-0.023219	0.03485	0.058069	3.7e-05	0.01135
original_gldm_SmallDependenceLowGrayLevelEmphasis	-0.012783	0.038753	0.051537	-0.000459	0.010506
original_shape_MajorAxisLength	-0.018997	0.029599	0.048596	-0.000697	0.007056
original_glszm_ZoneEntropy	-0.006572	0.038704	0.045276	-0.000404	0.008266
original_glszm_SizeZoneNonUniformityNormalized	-0.008226	0.030508	0.038734	-0.000297	0.003889
original_glrlm_ShortRunLowGrayLevelEmphasis	-0.009744	0.023239	0.032983	0.000487	0.006795
original_glszm_GrayLevelNonUniformityNormalized	-0.007757	0.010527	0.018283	0.000991	0.003214

First-order statistical features also played crucial roles in the model. High values of original_firstorder_Kurtosis and original_firstorder_Skewness were consistently associated with positive SHAP values. These findings are supported by quantitative CT texture analysis studies. Lee et al. demonstrated that kurtosis on CT texture analysis significantly correlates with the malignant risk grade of GISTs, and achieved an area under the curve of 0.779 for differentiating high-risk from low-risk GISTs (69). These statistical characteristics—sharper intensity peaks and asymmetric intensity distributions—may reflect intratumoral heterogeneity, including necrosis, hemorrhage, or cystic degeneration, which are frequently observed in larger or higher-risk GISTs (69).

Texture features displayed distinct influence patterns. Certain features derived from the Gray Level Size Zone Matrix (GLSZM) and the Gray Level Dependence Matrix (GLDM), such as original_glszm_LargeAreaLowGrayLevelEmphasis and original_gldm_SmallDependenceLowGrayLevelEmphasis, showed that their lower values (indicated by blue point clusters) were associated with negative SHAP values. This observation aligns with known CT characteristics of gastric cancer, which often demonstrate more homogeneous enhancement patterns compared to GISTs. Additionally, Lee et al. reported that tumor heterogeneity on CT is a significant predictor of high-risk GISTs, suggesting that the absence of heterogeneous texture patterns may characterize non-GIST lesions. This finding enhances our understanding of morphological and statistical features, creating a multidimensional framework that helps the model differentiate between the two types of lesions.

**Figure 8:**
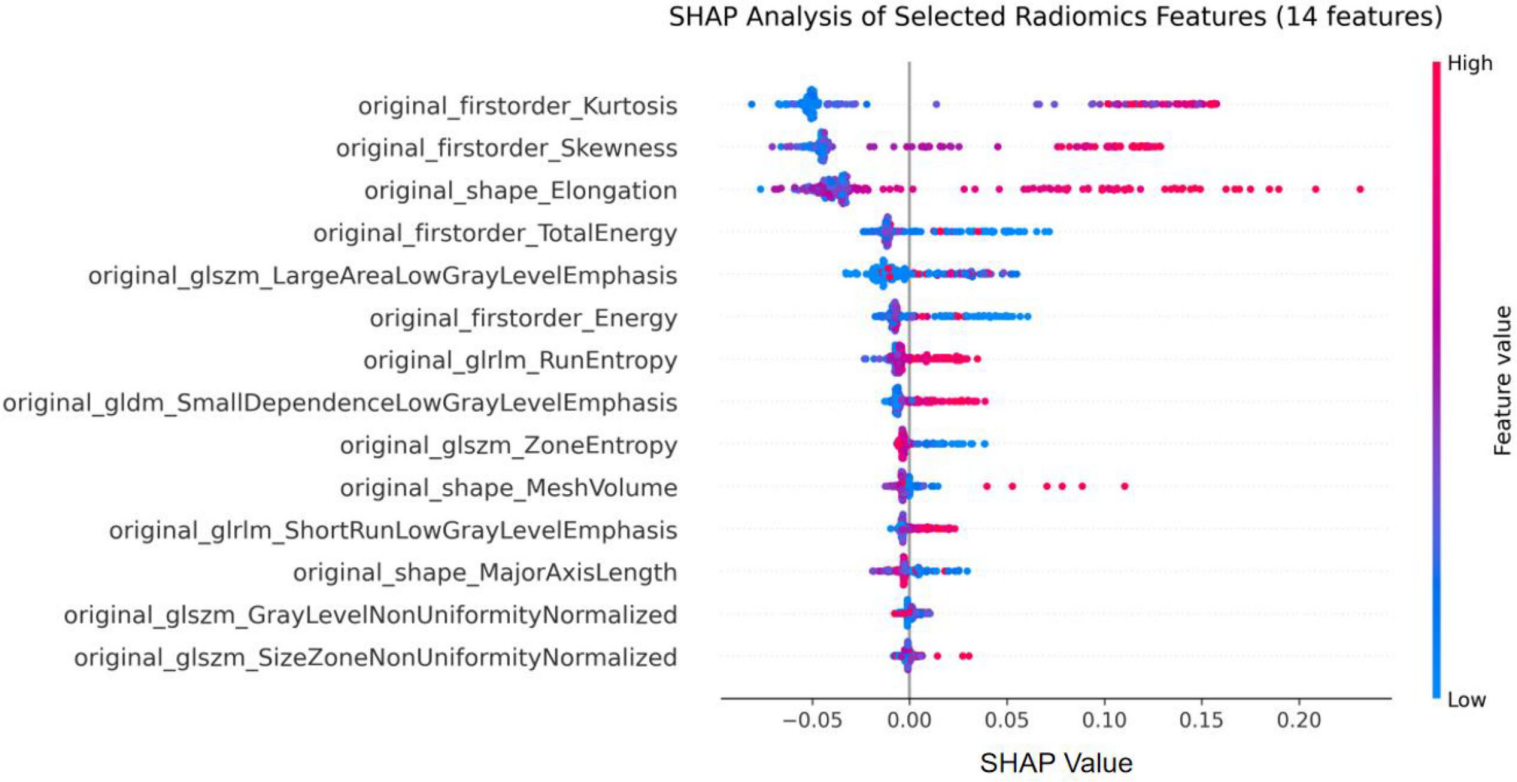
SHAP analysis of radiomics features.

As shown in **Figure 8**, the SHAP summary plot visualizes these complex relationships. Each point in the plot represents a single instance of a feature from a sample. The horizontal position of a point indicates both the direction and magnitude of that feature’s impact on the prediction, while its color (red or blue) represents the relative magnitude of the feature value itself. The overall width of the point clouds along the horizontal axis gives an intuitive understanding of the variance in influence among different features. Additionally, the color distribution along the horizontal axis reveals whether there are simple or complex relationships between feature values and prediction outcomes. For example, the noticeable shift of red points to the right for “original_shape_Elongation” supports the intuitive idea that greater elongation increases the likelihood of a GIST diagnosis.

This SHAP-based interpretability framework effectively connects high-dimensional radiomic signatures with clinically understandable decision-making. By quantifying and visualizing the contribution of each feature to the final prediction, the model shifts from being an opaque “black-box” classifier to an explainable diagnostic assistant. Specifically, clinicians can track how factors such as high elongation (suggesting exophytic GIST growth), elevated kurtosis (correlating with higher risk grade), and low large-area low-gray-level emphasis (characterizing non-GIST lesions) combine to influence the model’s classification. Importantly, recent studies have demonstrated that interpretable machine learning models incorporating radiomics features can effectively predict Ki-67 expression in GISTs, with SHAP analysis identifying radiomics scores and tumor diameter as the most influential contributors (AUC up to 0.840) (70). These linkages provide testable hypotheses for future studies correlating specific radiomic features with histopathological markers such as mitotic index, Ki-67 proliferation index, or KIT mutation status. This level of interpretability not only validates the model’s internal consistency but also supports its integration into clinical workflows, where understanding the reasoning behind a prediction is just as important as the prediction itself.

##### 4.3.4.4 Analyze the impact of the clinical feature

This study employed the SHAP (Shapley Additive Explanations) method to quantitatively evaluate the contribution of four clinical features, namely tumor area, location, sex, and age, to the prediction of GISTs. SHAP values were computed for each feature to systematically assess their relative importance in the model’s output.

**Figure 9:**
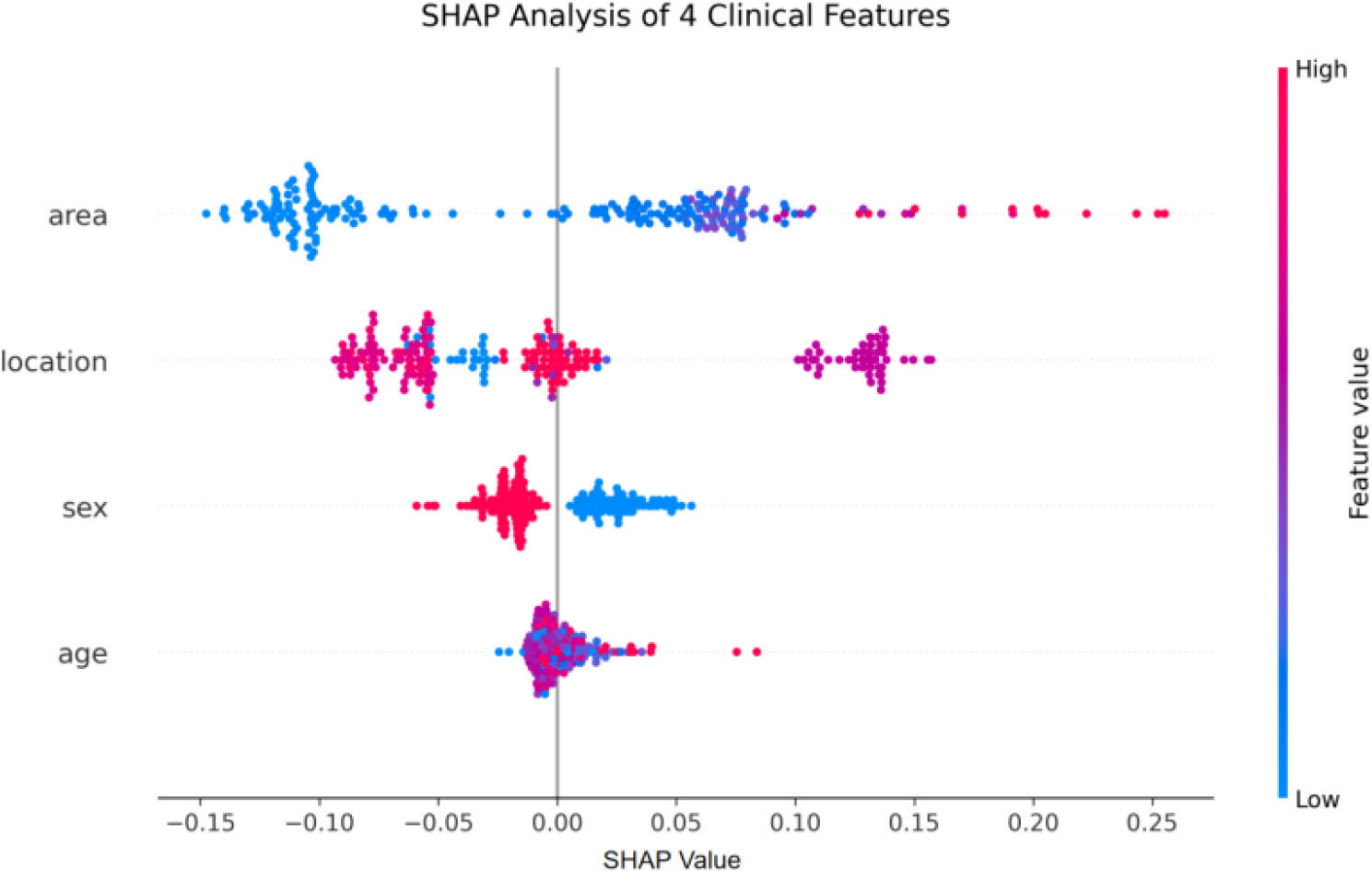
SHAP analysis of clinical features.

The summary plot of SHAP values is shown in **Figure 9**, and the corresponding summary statistics are shown in **Table 11**, and the results illustrate the hierarchy of feature importance and their directional effects. Among the features, “Area” displayed the widest range of SHAP values (range from −0.1475 to 0.2552), indicating its strong influence on GIST prediction. Smaller areas (represented by blue dots) were associated with negative SHAP values, corresponding to a decreased probability of GIST. In contrast, larger areas (red dots) clustered in the positive SHAP value region, indicating an increased likelihood of GIST. This pattern aligns with clinical observations that GISTs often present as larger submucosal masses.

**Table 11:** Descriptive statistics of SHAP values for clinical features.

Feature	Min	Max	Range	Mean	Std
Sex	-0.059075	0.056340	0.115415	-0.000967	0.024884
Age	-0.024478	0.083837	0.108315	0.000660	0.012844
Location	-0.093333	0.157133	0.250466	-0.002017	0.076401
Area	-0.147459	0.255225	0.402684	0.000219	0.097293

The “Location” feature, with SHAP values ranging from −0.0933 to 0.1571, also played a notable role. Anatomical sites were encoded numerically (1: cardia, 2: gastrofundal junction, 3: abdominal cavity, 4: esophagus, 5: gastroantral junction, 6: fundus, 7: antrum, 8: gastric angle, 9: body, 10: pylorus). Locations marked by red dots in the positive SHAP region were associated with higher GIST probability, whereas those with blue dots in the negative region indicated lower risk, reflecting site-specific susceptibility.

For the “Sex” feature (SHAP range from −0.0591 to 0.0563), female patients (encoded as 0) were predominantly associated with positive SHAP values, suggesting a moderately increased probability of GIST. Male patients (encoded as 1) tended to correspond to negative SHAP values, indicating a relatively lower risk. This points to possible sex-based differences in GIST occurrence, though the underlying factors require further study.

The “Age” feature showed the narrowest SHAP value range from −0.0245 to 0.0838, implying a comparatively modest influence. Although most samples clustered near zero, older age (red dots) was linked to slightly elevated SHAP values, consistent with the higher incidence of GIST in older populations.

In summary, “Area” was identified as the most influential feature in distinguishing GIST from non-GIST, followed by “Location” and “Sex,” while “Age” had a more limited effect. These results offer interpretable insights into the clinical determinants of GIST prediction.

#### 4.3.5 Discussion

This study aimed to develop classification models for gastric tumors using deep learning and radiomics based on EUS images. Single-parameter models were constructed using deep learning, radiomic, and clinical features separately, while a multi-parameter model was built using decision-level fusion. The combined model was developed and evaluated on a group of 219 patients. It showed improved performance compared to the single-parameter models, achieving an accuracy of 86.36%, a sensitivity of 100.00%, a specificity of 82.86%, and an area under the curve of 0.95.

To address the class imbalance in our dataset (with a GIST to non-GIST ratio of approximately 1:4), we implemented a data-driven threshold optimization strategy instead of using the default threshold of 0.5. By maximizing Youden’s index on the training set, we determined an optimal threshold of 0.2, which was applied consistently across the dataset. This approach aimed to enhance clinical utility by balancing sensitivity, emphasizing the detection of true GIST cases, with specificity, rather than solely focusing on overall accuracy. The result was perfect sensitivity (100%) on the test set, albeit with a slightly moderated specificity of 82.86%. This demonstrates the effectiveness of our strategy for clinical applications, particularly in reducing false negatives during the initial screening of gastric submucosal tumors, where such precision is crucial.

The enhanced performance of the combined model can be attributed to the complementary nature of the three feature types. Radiomic features provide quantitative descriptions of tumor texture, shape, and intensity, which help characterize tumor morphology. Deep learning features, extracted via CNNs, capture high-level abstract patterns that may not be evident through conventional radiomic analysis. Clinical features, including patient demographics and tumor characteristics, offer valuable contextual information for classification. The integration of these multimodal features allows the model to comprehensively assess tumor heterogeneity, thereby improving diagnostic performance.

In our feature selection strategy, we used a two-stage approach that combined t-tests and LASSO regression. To validate this approach, we compared its performance with two alternative methods: mutual information and recursive feature elimination. Our t-test plus LASSO method achieved the highest AUC (Area Under the Curve) and accuracy among the three methods, demonstrating a perfect sensitivity of 1.00. Although mutual information produced comparable results using a more concise feature set, our method outperformed it in terms of AUC and accuracy. In contrast, recursive feature elimination displayed significantly lower sensitivity, highlighting its limitations in identifying positive cases. The success of the t-test and LASSO combination can be attributed to their complementary strengths: the t-test acts as an effective univariate filter to eliminate irrelevant features, while LASSO performs joint feature selection through L1 regularization, addressing multicollinearity and reducing the risk of overfitting. This two-stage strategy has been widely adopted in radiomics studies. Additionally, the interpretability of LASSO coefficients facilitated subsequent SHAP (SHapley Additive exPlanations) analysis for clinical interpretation, aligning with recent approaches in multi-modal medical image analysis. Therefore, the t-test and LASSO combination represents a practical and well-validated choice for feature selection in our study. Regarding our choice of deep feature extraction architecture, we opted for a CNN-based autoencoder instead of a Vision Transformer. While transformers have proven successful in many computer vision tasks, they generally require large-scale pre-training data to learn generalizable representations. Our dataset, comprising 219 patients, is of moderate size and may not be sufficient for optimal training of a Vision Transformer. This limitation is evident in our comparative results, where the Vision Transformer achieved a sensitivity of only 0.56 on the test set, compared to 0.78 for the EfficientNet models. In contrast, our autoencoder, which leverages CNN-based inductive biases and can be pre-trained in an unsupervised manner, achieved an AUC of 0.95 and a sensitivity of 1.00. Therefore, we conclude that CNN-based autoencoders are a practical and effective choice for classifying gastric tumors based on endoscopic ultrasound, particularly in data-constrained clinical settings.

Our results align with recent studies highlighting the advantages of combining radiomics and deep learning in medical image analysis. For example, Ye et al. (71) reported that integrating radiomic and deep learning features improved the classification of lung adenocarcinoma, achieving an AUC of 0.898. Similarly, Ning et al. (72) noted limitations of single-modality approaches, particularly in detecting subtle imaging patterns in gastric tumors. Many existing methods rely predominantly on single-modality features, which may offer partial tumor information but often fail to capture their full complexity, missing subtle yet critical characteristics. Although deep learning models can learn high-level features, they frequently lack interpretability, which is important in clinical contexts where understanding model decisions is essential. The proposed combined model addresses these limitations by integrating radiomic, deep learning, and clinical features. Analyzing gastric tumors from multiple perspectives represents a step forward in EUS-based classification, offering a more accurate and reliable tool for clinical diagnostics.

Endoscopic ultrasonography images used for feature extraction have distinct advantages. This non-invasive imaging modality is widely utilized in clinical practice due to its straightforward operation and rapid imaging capabilities. EUS can produce high-resolution images of the gastrointestinal tract wall and adjacent structures in real-time, greatly supporting the early detection and precise analysis of gastric tumors. The model developed from these imaging techniques can be seamlessly integrated into clinical workflows, offering a practical and efficient solution for identifying GISTs.

A critical factor for the clinical application of our model is the context-dependent interpretation of its predictive values. The positive and negative predictive values reported in this study are based on the prevalence of GIST in our specific cohort, which is approximately 19.6%. These metrics are intrinsically linked to disease prevalence, as described by Bayes’theorem. Therefore, when using this model in a different clinical setting with a different pre-test probability of GIST, the PPV and NPV must be recalculated using the model’s fixed sensitivity and specificity (at the 0.2 threshold) along with the local prevalence estimate.

This study has several limitations. Firstly, and most significantly, the model was trained and validated using a dataset from a single center. The lack of an independent multicenter external validation cohort greatly restricts the generalizability of our findings. Moreover, to the best of our knowledge, there is currently no publicly available EUS dataset with matched histopathology labels for gastric tumors, which precludes external benchmarking on a public repository at this stage. This limitation includes potential variations in imaging protocols and patient demographics, which can directly impact the clinical interpretation of predictive values. The stable performance metrics of the model need validation in different settings, and the PPV and NPV will inherently vary based on local disease prevalence. It remains uncertain how the model would perform with data from other hospitals, different EUS systems, or different operators, as variations in scanning protocols and image characteristics are expected. Additionally, there is a concern about overfitting to center-specific patterns. Secondly, the independent test set consisted of only 44 patients, which is relatively small. While our training and validation sets included 4,806 images from 219 patients, the standalone test set used for final evaluation only had 44 patients. This raises legitimate concerns regarding the generalizability and robustness of the proposed model, as highlighted in similar deep learning studies that faced challenges due to limited sample sizes. For example, Bhatt et al. (73) noted issues of data imbalance and overfitting when working with restricted clinical datasets, while Pauranik et al. (74) addressed the problem of having too few endoscopic images for gastric cancer detection. Thirdly, although the combined model shows improved performance, there may be opportunities for further enhancement through advanced network architectures or the inclusion of additional clinically relevant variables. To address these limitations within the constraints of our available data, we implemented several strategies. We adopted a strict patient-wise data splitting approach to prevent data leakage and create a more realistic evaluation scenario. We performed data augmentation to increase variability in training. We applied LASSO-based feature selection to mitigate overfitting by choosing only the most robust radiomic features and conducted feature importance analysis to identify the most influential clinical and radiomic features affecting model decisions. We also conducted comprehensive ablation studies to evaluate the contribution of each feature modality and optimized the classification threshold based on Youden’s index to balance sensitivity and specificity despite class imbalance.

This study offers valuable insights into the integration of multimodal features for classifying gastric tumors. The combined model effectively distinguishes between GIST and non-GIST cases, aiding clinicians in making more informed diagnostic decisions. By providing an objective, data-driven approach, this model has the potential to serve as a useful tool in the clinical management of gastric tumors, ultimately benefiting patient care.

Future research should focus on several key areas to advance this work. First and foremost, it is essential to conduct external validation with multi-center, prospective cohorts to definitively establish the model’s broad clinical applicability and robustness. Collaborations with institutions that utilize different EUS platforms are planned to initiate this important step. Additionally, future studies could investigate the model’s applicability in various clinical settings by using expanded datasets. There is also the potential to develop integrated decision-support systems to enhance the intelligent and precise diagnosis of gastric tumors.

## 5 Conclusion

The proposed model integrates radiomic features, deep learning features, and clinical attributes to classify gastric tumors as GISTs and non-GISTs using endoscopic ultrasonography images. By integrating these diverse features, the model enhances our understanding of gastric tumors and improves tumor classification. This approach can assist clinicians in identifying GISTs and provides valuable diagnostic support.

## Data Availability

The original contributions presented in the study are included in the article/supplementary material. Further inquiries can be directed to the corresponding authors.
